# Supplementary material for: Coral-associated bacteria demonstrate phylosymbiosis and cophylogeny
Source: Nat Commun. 2018 Nov 22;9:4921. doi: 10.1038/s41467-018-07275-x (PMC6250698; doi:10.1038/s41467-018-07275-x)
Supplement: Supplementary file 3 — Supplementary Information [file 41467_2018_7275_MOESM3_ESM.pdf]

Supplementary Information

Pollock *et al.*, "Coral-associated bacteria demonstrate phylosymbiosis and cophylogeny"

Supplementary Note 1

Sequencing depths Following quality control, removal of chimeras and singleton sequences, and exclusion of environmental and outgroup libraries from the main analysis (except where otherwise noted), 649 scleractinian coral 16S rRNA gene amplicon libraries remained. Of these, 30 samples had fewer than 1000 sequences/sample, 80 samples had 1000-5000 sequences/sample; 112 samples had 5000-10000 sequences; 141 samples had 10000-15000 sequences; 294 samples had 15000-20000 sequences; 131 samples had >20000 sequences. To prevent unequal sequencing depths from influencing the analysis, we rarified to 1000/sequences per sample, which gave 619 total coral mucus, tissue, and skeleton samples. However, we also tested 5000, 10000, 15000, or 20000 sequences per sample for alpha-diversity and core-microbiome analysis. For differential abundance testing, we used 1000 sequences/sample rarefaction, or a parametric method within the phylogenetic GLMMs to account for sequencing depth variation.

Supplementary Note 2

A brief overview: dominant phyla in the coral microbiome Before comparing differences between compartments at more detailed taxonomic levels, we summarized the proportional representation of bacterial and archaeal phyla in the coral microbiome, averaged across all samples. At the coarsest scale, Proteobacteria were by far the most abundant bacterial or archaeal phylum in the dataset (56.5% of total reads), ~4-fold more abundant than the next most abundant phylum (Bacteroidetes, 14.2%), and ~45-fold more abundant than the most abundant archaeal phylum (Crenarchaeota, 1.3%). Like many host-associated microbial communities, coral microbiomes were highly uneven: 10 phyla accounted for 90% of the microbiome, averaging across all samples (Proteobacteria, 56.5%; Bacteroidetes, 14.2%; Firmicutes, 5.7%; Cyanobacteria, 4.4%; Actinobacteria, 3.5%; Planctomycetes, 1.7%; Crenarchaeota, 1.3%; Chlamydiae, 1.1%; Chloroflexi, 1.1%; Verrucomicrobia, 0.8%), with 59 other phyla, each at <1% mean abundance, making up the remainder. Even after removing identifiable mitochondria and chloroplasts, an average of ~4.9% of reads in each sample could not be assigned to a phylum.

Supplementary Note 3

Microbiome richness differs between coral mucus, tissue, and skeleton Microbiomes from distinct anatomical compartments differed significantly in OTU richness (i.e., observed OTUs per 1000 reads) (df = 2, F-value = 19.55, $p < 0.001$; Fig. 1a). The coral mucus layer hosted significantly lower richness (119.6 ± 6.8l OTUs per 1000 reads) than coral tissue (166.5 ± 11.1; $p = 0.0015$) and skeleton (208.7 ± 11.9; $p < 0.001$) (Fig. 1a). OTU richness of endolithic communities within coral skeletons was 75% higher than coral mucus ($p < 0.001$) and 25% higher than tissue ($p = 0.010$) (Fig. 1a). These differences were also significant at rarefaction depths of 5000, 10000, or 15000. At 20000 sequences per sample, all compartment differences

were significant except mucus vs. tissue, likely due to removal of most tissue samples at this depth (n = 17 vs. n = 199 at 1000 sequences per sample).

Supplementary Note 4

Compositional differences between compartments are robust to choice of distance metric
As expected, given the complexity of underlying variation in coral phylogeny, environment, and anatomy, the first 3 PC axes captured only a small fraction of overall variation in community distances (PC1 8.9%; PC2 4.3%; PC3 2.1%). However, statistical analyses of all multivariate dissimilarity measures examined (Bray-Curtis, Weighted UniFrac and Unweighted UniFrac) revealed significant differences in microbial community composition among coral compartments (all Adonis $p < 0.001$, Fig. 1b). Microbial communities from different compartments separated along the second principal coordinates axis, with mucus samples clustering towards the positive end of this axis, transitioning to tissue and then to skeleton microbiomes near the negative end of the axis (Fig. 1b).

Supplementary Note 5

Coral mucus, tissue, and skeleton host distinct core microbiomes 16S rRNA V4 amplicons sequenced from scleractinian coral mucus, tissue and skeleton (n = 614) were evaluated for differences in 'core' microbiome membership, community composition and diversity, and the influence of environmental and host conditions on these parameters.

Core microbiome membership was assessed at multiple taxonomic levels by identifying taxa that were present in more than 70% of rarefied samples within a given compartment. At the OTU level, no specific bacterial or archaeal OTUs were core members of any compartment at 1000 reads per sample, with the exception of a single OTU of water-column *Synechococcus* consistently associated with mucus microbiomes. Note that at 1000 reads per sample, a relatively rare microbe with 0.5% abundance will be sequenced in 2 or more reads ~95.9% of the time (cumulative binomial, p=0.005, n=2, trials=1000). Microbes that are biologically part of the core microbiome, but not observed here are thus likely to have low abundances. This shallow depth, which includes the greatest number of biological samples, was therefore deemed sufficient for most of our analyses (which interrogate the ecology and evolution of the main lineages of bacteria and archaea in each compartment).

We also calculated core microbiomes at higher read depths. At 5000 seqs/sample, a microbe with an abundance of 0.05% will be detected with 2 reads in ~71.3% of samples (cumulative binomial p=0.0005, n=2, trials=5000). At this depth, results were identical as at 1000 seqs/sample, except that *Endozoicomonas* OTU 739464 was consistently associated with tissue microbiomes.To test whether broader taxonomic ranks were conserved, we also calculated which bacterial orders were prevalent across all samples with 1000 reads or more. All compartments shared 6 core microbiome members: Bacteroidetes from the order Flavobacteriales*;* unclassified α-Proteobacteria and α-Proteobacteria from orders Rhizobiales and Rhodobacterales; and γ-Proteobacteria from the orders Oceanospirallales and Alteromonadales. In addition, compartments had unique core microbial orders: the Cyanobacteria order Synechococcales and the α-Proteobacteria order Rickettsiales (both common in coastal waters) were core in mucus;

Clostridiales and Rhodospirillales were core in skeleton; and Cytophagales were core in both tissue and skeleton.

Together, these results indicate a pattern of conservation in the orders of bacteria present in coral microbiomes, but variability at finer taxonomic scales. While the core microbiome (i.e. OTUs with >70% prevalence) of coral mucus and tissue held only *Synechococcus* in mucus and *Endozoicomonas* OTU 739464 in tissue, several bacterial orders were consistently present in coral mucus, tissue, and skeleton, even across very diverse coral hosts spanning large geographic ranges. Greater consistency of microbial orders than OTUs could be due to partial niche overlap between OTUs within the same order, or may reflect co-diversification of some strains of bacteria with their host.

Supplementary Note 6

Overlapping prevalent microbial orders in Australian and Florida Keys Coral Mucus We tested whether the microbial orders identified as highly consistent in this study were consistent with past results. In a time-series study of the mucus microbiome of corals exposed to simulated overfishing or nutrient pollution, Zaneveld *et al.* calculated core orders in the coral mucus of three genera of corals in plots in the Florida Keys[2]. 100% of the 8 orders of bacteria found in >70% of coral mucus samples in this study were also found to be prevalent (>95% of samples) in coral mucus from Zaneveld et al., 2016. (The converse was not true: only 8/11 orders with >95% prevalence in control corals from Zaneveld et al., 2016 were highly prevalent here). Similarly, OTUs belonging to 7/8 of these named orders (Synechococcales, Oceanospirallales, Alteromonadales, Rickettsiales, Rhodobacterales, Rhizobiales, and Flavobacterales, but not unclassified α-Proteobacteria) were part of the core microbiome in Apprill et al., 2017[1].

Supplementary Note 7

Confirmation of *Candidatus* Amoebophilus as a common coral associate

Apprill *et al.*,[1] previously identified *Candidatus* Amoebophilus as a core member of the microbiome of three coral species. This finding was intriguing because *Candidatus* Amoebophilus are intracellular symbionts of microbial eukaryotes, so we decided to look for it in our data. Using the same definition of 'consistent' association (presence in >50% of samples) and when rarefying at an equal read depth (10,000 sequences/sample) we also find *Candidatus* Amoeobophilus (specifically Greengenes OTU 321533 with taxonomy string 'k__Bacteria; p__Bacteroidetes; c__Cytophagia; o__Cytophagales; f__[Amoebophilaceae]; g__SGUS912;s__') to be one of 11 OTUs present in the 50% tissue 'core microbiome' at 10000 sequences/sample.

Supplementary Note 8

Coral compartments differ in consistency. We calculated two measures of the consistency of coral compartments: the proportion of reads that were part of 'core' microbial orders (present in 70% of samples), and inter-colony variation in beta-diversity. The proportion of reads belonging to core microbial orders differed significantly among compartments (df = 2, F-value = 27.2, $p < 0.001$; Supplementary Figure 2a). The microbiome of coral mucus, which is in closest proximity to surrounding seawater, had higher core microbiome abundance than tissue or skeleton. Microbes belonging to core orders accounted for 64.5 ± 1.7% of reads within mucus microbiomes, 30% more than in tissue (49.7 ± 2.0%; $p < 0.0001$) and 38% more than in skeleton (46.7 ± 1.8%; $p < 0.0001$; Supplementary Figure 2a). Similarly, overall community composition was least variable in mucus across samples (Weighted UniFrac distance, $p < 0.001$; Supplementary Figure 2b). Thus, on both measures, mucus microbiomes were more consistent in composition than tissue or skeleton microbiomes. This may reflect that, despite some species differences, variation in mucus microbiomes between coral species is lower than tissue or skeleton microbiomes.

Supplementary Note 9

In healthy corals, host species has a stronger influence on the microbiome than geography. To assess the relative influence of host and environmental factors on coral microbiomes, the impacts of host (e.g. host genus and disease susceptibility) and environmental (e.g. collection season, reef, and latitude) variables were quantified for mucus (n = 207), tissue (n = 199), and skeleton (n = 208) microbiomes (OTU level) across scleractinian coral genera. Several host and environmental factors significantly influenced microbial community composition in each of the three coral compartments (all Weighted UniFrac Adonis $p < 0.05$ in all compartments; Fig. 1c). Across all compartments, host species had the greatest influence on microbial community composition (raw R^2 0.37-0.48), and in tissue explained nearly half the variance in community composition. Host species was still the most influential variable after adjusting R^2 values for the degrees of freedom in each variable (adjusted R^2 0.15-0.24). In the context of this study, sampling location (reef name) explained less variance than host species (raw R^2 0.16-0.20) and had consistently lower adjusted R^2 values than host species. However, the relative influence of these and other measured parameters differed among compartments.

Supplementary Note 10

Across all compartments and multivariate dissimilarity measures, more specific taxonomic ranks explain more microbiome clustering. To test the explanatory power of various levels of coral taxonomy, we ranked the Adonis adjusted R^2 value of coral species, genus, clade *sensu* Fukami (family-level group), and complex vs. robust clade membership (broadest division). A table of these values is reported in Supplementary Data 3. The absolute R^2 values differed across compartments, metrics, and rarefaction depths (e.g. higher R^2 values for host factors in tissue). However, their relative rank was remarkably consistent. Across all compartments, three dissimilarity measures (Weighted UniFrac distance, Unweighted UniFrac distance, and Bray Curtis dissimilarity) and two rarefaction depths (1000 sequences/sample or 10000 sequences/sample), more specific taxonomic levels always explained microbiome beta-diversity better than more general ones. The largest fall in explanatory power typically occurred between genus and family-level group, and between family-level group and Complex vs. Robust clade membership. This analysis is largely consistent with signals of phylosymbiosis in Mantel test results, and also shows that the relative effect of coral taxonomy on microbiome membership is robust to common choices for rarefaction depth and dissimilarity measure.

Supplementary Note 11

Host factors more strongly influence coral tissue and skeleton microbiomes while environmental conditions more strongly influence mucus communities. The influence of host factors on microbiome composition (i.e., Weighted UniFrac Adonis R^2) was most pronounced in the tissue and skeleton compartments, whereas environmental factors tended to have the strongest influence on mucus communities (Fig. 1c). Similarly, host genus had the strongest influence on tissue microbiomes. The influence of host genus on tissue microbiome composition was 1.53-fold greater than on mucus microbiome composition (mucus raw Adonis $R^2 = 0.249$; tissue raw $R^2 = 0.380$) and 1.14-fold greater in skeleton microbiomes (raw $R^2 = 0.334$; Adonis Bonferroni $p < 0.05$ in all compartments; Fig. 1c). Microbiome composition was also significantly correlated with disease susceptibility (i.e., 10-year genus-level disease prevalence on mid-shelf reefs of the northern Great Barrier Reef; Willis Great Barrier Reef Disease Database v20161016[3]), and like other host-associated factors species-wide disease prevalence was most strongly correlated with microbiome composition in tissue and skeleton (Adonis Bonferroni $p < 0.05$; Fig. 1c). Conversely, reef (i.e., collection site) also influenced coral mucus ($R^2 = 0.209$) 1.15-fold more strongly than coral tissue ($R^2 = 0.184$) and 1.28-fold more strongly than skeleton communities ($R^2 = 0.163$) (Weighted UniFrac Adonis Bonferroni $p < 0.05$ in all compartments; Fig. 1c). Latitude had a small but consistent effect on community composition. Latitude was significantly correlated with microbiome structure in all compartments (all Adonis $p < 0.05$; Fig. 1c), and again this environmental factor had the strongest effect on communities in the mucus. (mucus $R^2 = 0.045$; tissue $R^2 = 0.020$; skeleton $R^2 = 0.018$; Fig. 1c; Supplementary Data S3). Finally, we conducted a separate analysis of the effects of sampling season (i.e., summer vs. winter) using only the subset of samples collected at Lizard Island. Like other environmental parameters, sampling season influenced mucus microbial communities ($R^2 = 0.108$) 3.29-fold more strongly than tissue ($R^2 = 0.033$) and 2.78-fold more strongly than skeleton communities ($R^2 = 0.039$) (Adonis $p < 0.05$ in all compartments; data for the Lizard island subset used in this analysis not shown).

Together, these data show that for healthy Australian corals, host species tended to have a stronger influence than geography or measured environmental parameters in all compartments tested (i.e. including mucus). They also show that host traits tend to have a stronger relative influence on coral tissue and skeleton microbiomes relative to mucus, whereas environmental parameters have a stronger relative influence on mucus microbiomes compared to tissue or skeleton.

Supplementary Note 12

Putatively opportunistic bacteria associated with small corals We tested how relative coral size influenced the microbiome. Several measures of relative coral colony size showed significant correlations with microbiome composition. We tested both absolute coral dimensions as well as those sizes normalized by the largest dimensions recorded for that coral in this study (prop_Colony_maximum_GCMP_recorded) or in either this study or the coral traits database (prop_Colony_maximum_universal). Using either measure, tissue and skeleton microbiomes were significantly associated with coral size (raw Adonis $p < 0.05$). Following stringent Bonferroni correction across all factors and compartments, the association between coral colony size and microbiome composition was significant only in coral skeleton (Adonis permutational $p < 0.05$), though even there the magnitude of the effect was very small (raw $R^2 <= 0.022$). Phylogenetic GLMMs showed that 14.9% (51/343, mucus), 47.6% (214/450, tissue), and 31.2% (151/483, skeleton) of genera were significantly associated with smaller corals; while 7.3% (25/343, mucus), 2.7% (12/450, tissue), and 7.5% (36/483, skeleton) were significantly associated with larger corals. Additionally, pGLMMs showed that *Balneola* was significantly less abundant in large corals in both the tissue (upper 95% CI = -1.51) and skeleton (upper 95% CI = -0.22) microbial communities, and the abundance of unclassified Aurantimonadaceae was significantly lower in the skeletons of large colonies (upper 95% CI = -0.12), although its overall prevalence was too low in tissues to be tested.

Supplementary Figures

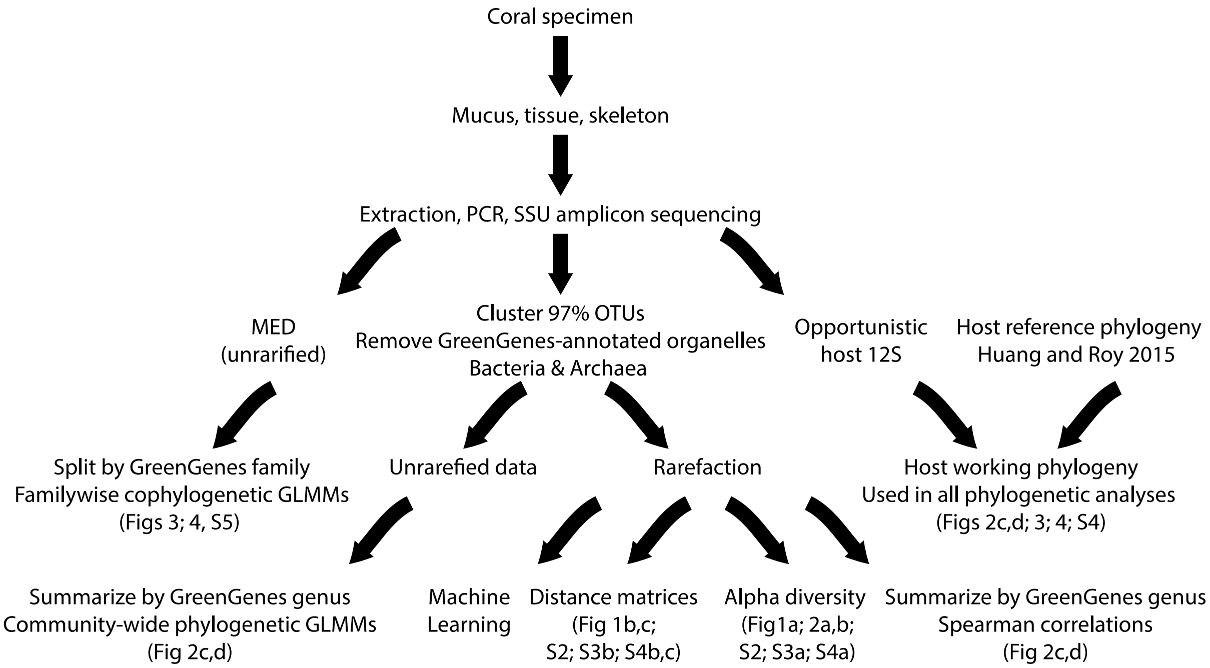

Supplementary Figure 1. Data processing workflow. Several sampling procedures, bioinformatics, and analytical methods were combined and used to evaluate our microbiome and coral host phylogeny data. All samples underwent collections in the field where corals were subjected to mucus, tissue and skeleton separation (see Methods for details). These samples were placed in MoBio PowerSoil kits, frozen, and shipped back to OSU or Penn State for processing. DNA was extracted according to the manufactures recommendations, and 16S V4 amplicon libraries generated (see Methods for details). All amplicon data then underwent quality control parsing (see Methods) prior to further downstream analysis. Amplicons were then subjected to several analytics to address different questions about the influence of the host and environment on coral microbiomes (see flow chart).

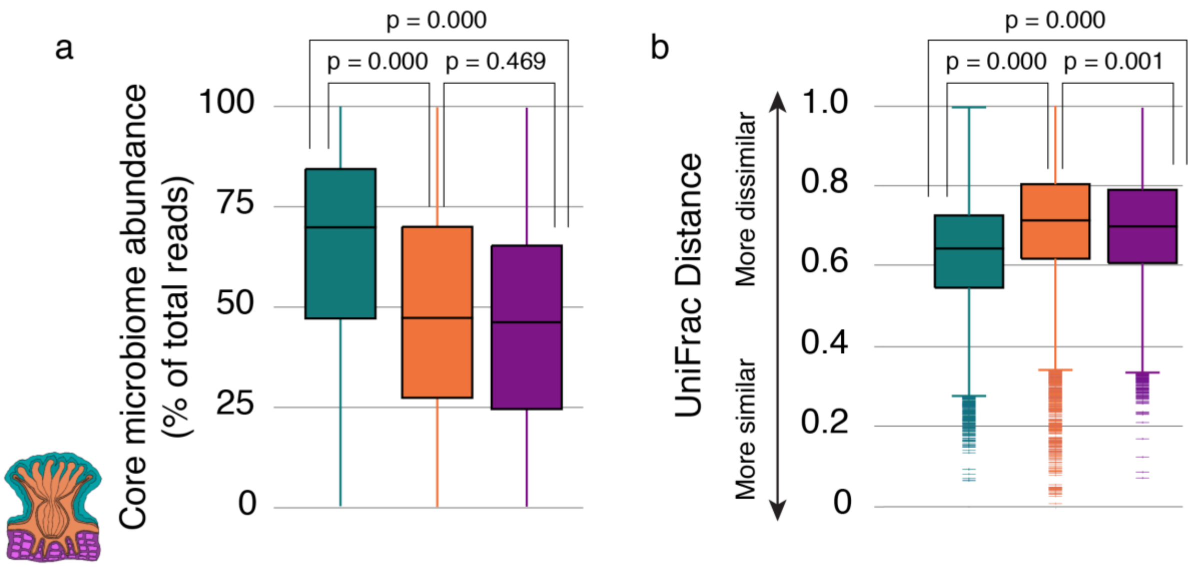

Supplementary Figure 2. **Microbiome consistency among coral compartments.** Compartments are denoted by color as mucus (teal), tissue (orange), and skeleton (purple). **a**) Core microbiome abundance. Bars show the proportion of sequence reads for each compartment that belonged to members of the core microbiome for that compartment. Microbial orders were deemed "core" if present in ≥ 70% of samples. **b**) Beta-diversity. Mucus compartments showed less microbiome variability than tissue and skeleton. All p-values reflect Tukey's HSD.

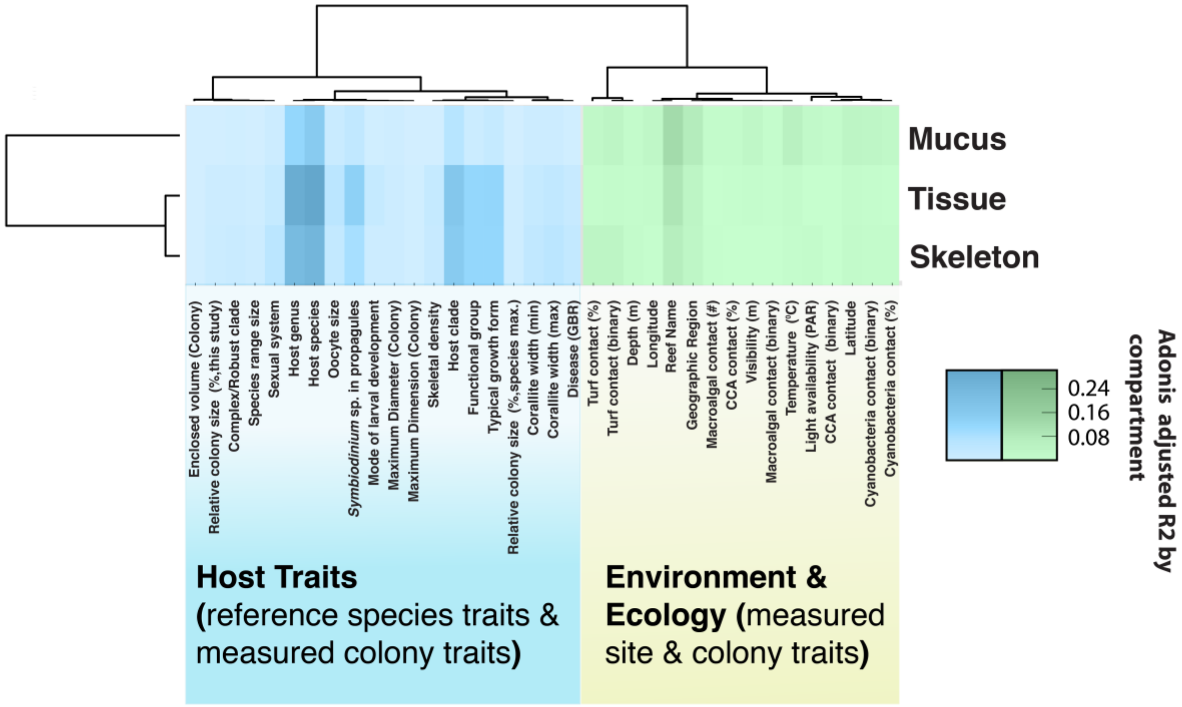

Supplementary Figure 3. **Influence of coral traits and local environment on microbial community composition.** This figure is presented for comparison with Fig 1c. The heatmap visualizes the influence of several host and environmental factors on microbial community composition in coral mucus tissue or skeleton. This figure is identical to **Fig. 1c** in the main text, except that it presents raw R^2 values, rather than R^2 values that are Z-score normalized within each factor. Thus, this figure is more useful for seeing which factors are influential in an absolute sense (e.g. even in mucus different host species have different microbiomes), while Fig 1c highlights the relative influence of each parameter across compartments (e.g. host species has a much stronger relative influence on tissue and skeleton microbiomes than on mucus). All the results in the figure are based on results from the Weighted UniFrac multivariate dissimilarity measure on data tables rarified to 1000 sequences/sample (see Supplementary Data S4 for alternative choices of dissimilarity measure or rarefaction depth). Light cells represent lower *Adonis* adjusted R^2 values for that factor (i.e. traits that have lesser influence on microbial community composition in a given compartment), whereas darker colors represent traits with a stronger influence. Traits were automatically clustered according to their R^2 values across compartments, and compartments were clustered according to the similarity of R^2 values within them. Host vs. environmental traits were manually colorized to highlight the split between host and environmental traits that emerged from clustering. Significance values for all trait x compartment combinations are available in Supplementary Data 4, and Bonferroni-corrected significance values for each combination are marked in Fig. 1c.

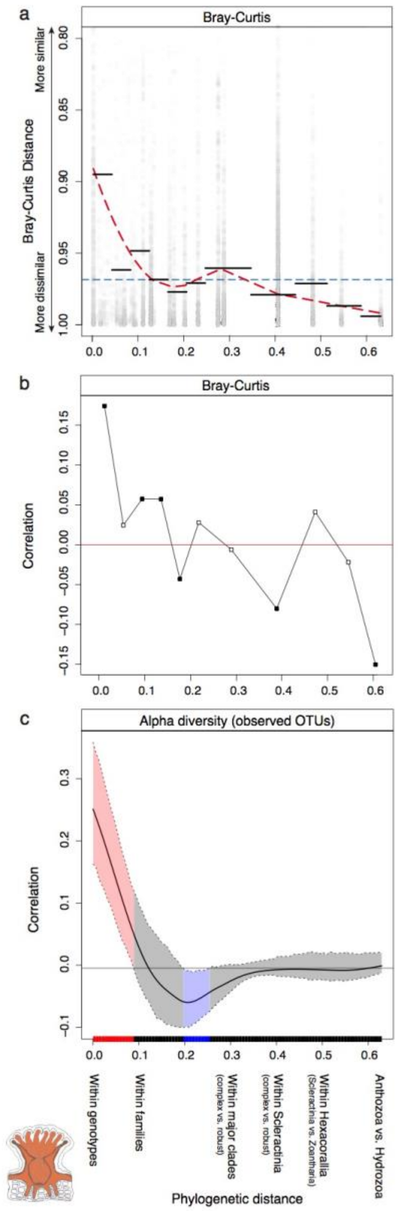

Supplementary Figure 4. **Phylogenetic correlograms of tissue microbiome diversity. a)** Scatterplot of phylogenetic distances versus Bray-Curtis dissimilarity. The grand mean of all pairwise community dissimilarities is shown as a dashed blue line, and the mean of community dissimilarities within each phylogenetic distance class is plotted as a horizontal black line throughout that class. A smoothed (loess) curve showing the overall trend in community dissimilarity is displayed as a dashed red line. **b)** Phylogenetic Mantel correlogram is shown for Bray-Curtis microbiome dissimilarity, with Mantel *r* versus coral host phylogenetic distance. Solid black boxes indicate phylogenetic distance classes within which pairwise microbial community dissimilarities are significantly different (i.e., significantly more similar or significantly less similar) from dissimilarities in all other distance classes (Mantel's *r*, $p < 0.05$), whereas open boxes indicate distance classes where dissimilarities are not significant. **c)** Correlogram showing autocorrelation in microbiome richness (Moran's *I* calculated from observed OTU values) versus coral host phylogenetic distance. Small phylogenetic distances indicate coral species of recent divergence. Red bars on the x-axis correspond to phylogenetic distances where microbial community parameters are significantly more similar between samples at a given distance class than between samples at all other phylogenetic distances, and blue bars correspond to distances where parameters are significantly less similar.

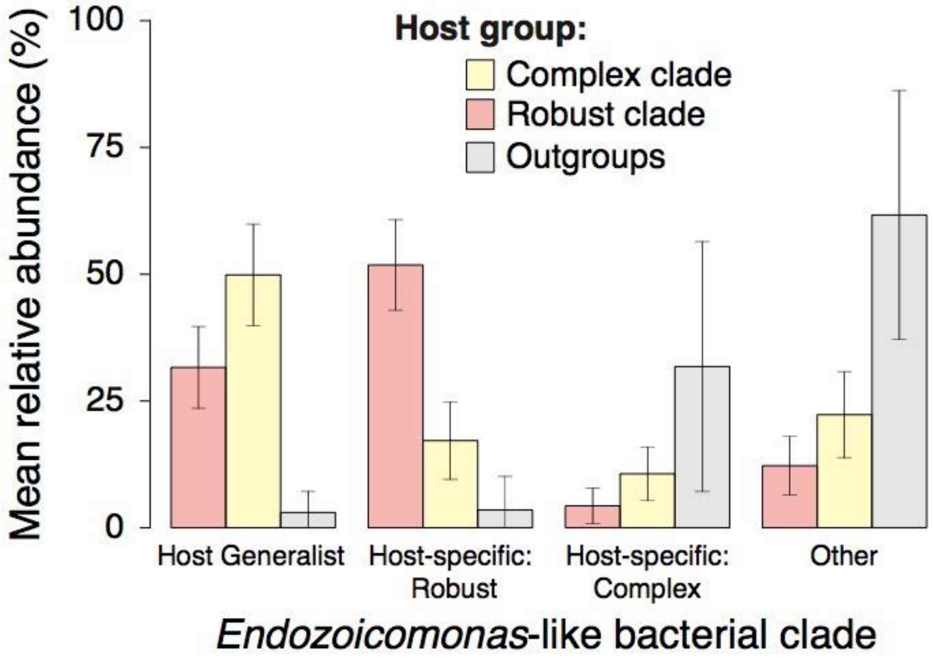

Supplementary Figure 5. Distribution of *Endozoicomonas*-like bacteria across Robust vs. Complex corals. The abundances of each *Endozoicomonas*-like bacterial clade (i.e., Host Generalist, Host-specific: Robust, and Host-specific: Complex, and others) in coral tissue samples are shown, relative to the total abundance of *Endozoicomonas*-like bacteria. Error bars indicate standard error ($n_{complex}$ = 90; n_{robust} = 124, $n_{outgroups}$ = 10).

Supplementary Data

Supplementary Data 1. **Sample Summary.** This Excel file summarizes samples collected and reefs visited in this study. This information is also available from the QIIME mapping file for samples (**Supplementary Data 2)**, but is summarized here for easier reference. **a.** Samples collected, subdivided by geographic region, coral species and coral compartment. **b.** Reefs visited as part of the study, along with longitude and latitude.

Supplementary Data 2. **Sample Metadata.** This Excel file is the QIIME mapping file containing all metadata used throughout the analysis.

Supplementary Data 3. **Prevalent 'Core' Microbes.** This Excel file summarizes prevalent microbes associated with coral microbiomes (e.g. 'core microbiomes', *sensu lato*) surveyed in this study, quantification of the effects of rarefaction depth on which OTUs are prevalent, and comparisons against two literature references. **a.** Graphical summary of microbial OTUs that had >70% prevalence at 1000, 5000, 10000, 15,000, or 20,000 sequences per sample. **b.** Machine readable data table of the prevalence and taxonomy of OTUs from panel a. **c.** Prevalence of microbial orders in the coral microbiome at 1000 seqs/sample, and a comparison of prevalent microbes in coral mucus with Zaneveld et al., 2016. **d.** Comparison with results from Apprill et al., 2016, conducted under similar rarefaction depth (10,000 seqs/sample) and prevalence threshold (50%).

Supplementary Data 4. Beta-diversity (multivariate dissimilarities). This Excel file provides a detailed accounting of factors influencing microbial β-diversity (multivariate dissimilarities or community composition) in each compartment according to several β-diversity metrics, and across rarefaction depths. **a.** Factors influencing microbiome β-diversity by compartment at 1,000 sequences per sample. **b**. Factors influencing microbiome β-diversity by compartment at 10,000 sequences per sample. **c.** Factors consistently and strongly associated with coral microbiome β-diversity by compartment at 1,000 sequences per sample. To find which factors were most consistently associated with microbiome beta-diversity, we calculated factors that were a) significant b) had adjusted R2 >= 0.05 for all distance metrics analyzed. **d.** Summary of how taxonomic ranks structure beta-diversity. Data from a and b are combined to illustrate that more specific taxonomic ranks for corals provide more information about microbial beta-diversity than more general ranks, regardless of distance metric and rarefaction depth chosen.

Supplementary Data 5. Alpha-diversity or richness. This Excel file provides a detailed accounting of microbiome richness. **a.** Results for permutational T-tests comparing coral microbiome vs. environmental community richness. Bacterial and Archaeal Diversity of Corals vs. Water and Sediment. Data reflect richness per 1000 reads of coral mucus, tissue, or sediment vs. reef water or sediment. **b.** Results for permutational T-tests comparing microbiome richness of corals vs. outgroups surveyed (blue corals, matt anemones, hydrozoans, etc).

Supplementary Data 6. Microbes correlated with host and environmental parameters. This Excel file summarizes microbes that were correlated with host and environmental parameters using either Spearman or Phylogenetic GLMM analysis. **a.** Spearman results summary. Summary of the number of bacterial genera significantly correlated with selected host or environmental metadata in each compartment, assessed by FDR-controlled Spearman regressions. For each factor, the Greeengenes taxonomy and R value for the top 3 genera positively or negatively correlated with that factor are listed. Additional statistical tests assess whether positive or negative associations are enriched for a given factor. **b.** Phylogenetic GLMM summary. Summary of numbers of genera associated with a subset of host and environmental factors, as identified in phylogenetic GLMMs. **c.** Full phylogenetic GLMM results. A comprehensive list of genera associated with a subset of host and environmental parameters, subdivided by tissue compartment, along with p values, estimated effect sizes, and 95% confidence intervals. For categorical data, the category value with which a microbial genus is associated is also reported.

Supplementary Data 7. Random Forest results. This Excel file describes coral host features that can be predicted from the microbiome. To measure the strength of association between the microbiome and coral physiology, we attempted to build supervised classification models using Random Forests analysis, and then back-predict certain host features. This addresses the question: "given microbial data alone, how much can you say about the coral host?". **a.** Results of Random Forest models of the coral microbiome, subdivided by compartment and the host trait predicted. **b.** Summary of host factors that can be accurately predicted from the coral microbiome, and which compartments predict them. Raw accuracies and error ratios are presented, and results with accuracy >70% and error ratios >1.0 are highlighted.

Supplementary Data 8. Mantel test results. This Excel file summarizes results from Mantel tests and Mantel correlograms (Methods). These permutation-based tests assess the degree of correlation between two distance matrices (e.g. geographic distance and genetic distance, etc). Here they were applied to test the extent to which host evolutionary distances corresponded to differences in microbiome composition, as reflected by between-sample beta-diversity distances. We calculated this measure for both a non-phylogenetic measure (Bray-Curtis divergences) and a phylogenetic beta-diversity distance metric (Weighted UniFrac distances).

Supplementary References

1. Apprill, A., Weber, L. G. & Santoro, A. E. Distinguishing between Microbial Habitats Unravels Ecological Complexity in Coral Microbiomes. *mSystems* **1,** e00143-16 (2016).
2. Zaneveld, J. R. *et al.* Overfishing and nutrient pollution interact with temperature to disrupt coral reefs down to microbial scales. *Nat. Commun.* **7,** ncomms11833 (2016).
3. Willis, B. L., Page, C. A. & Dinsdale, E. A. Coral disease on the Great Barrier Reef. in *Coral health and disease* (eds. Rosenberg, E. & Loya, Y.) 69–104 (Springer, 2004).
4. Darling, E. S., Alvarez-Filip, L., Oliver, T. A., McClanahan, T. R. & Côté, I. M. Evaluating life-history strategies of reef corals from species traits. *Ecol. Lett.* **15,** 1378–1386 (2012).
