# Supplementary material for: Coral-associated bacteria demonstrate phylosymbiosis and cophylogeny
Source: Nat Commun. 2018 Nov 22;9:4921. doi: 10.1038/s41467-018-07275-x (PMC6250698; doi:10.1038/s41467-018-07275-x)
Supplement: Supplementary file 4 — Description of Additional Supplementary Files [file 41467_2018_7275_MOESM4_ESM.docx]

**Description of Additional Supplementary Files**

File Name: Supplementary Dataset 1

Description: Sample Summary. This Excel file summarizes samples collected and reefs visited in this study. This information is also available from the QIIME mapping file for samples (Supplementary Data 2), but is summarized here for easier reference. a. Samples collected, subdivided by geographic region, coral species and coral compartment. b. Reefs visited as part of the study, along with longitude and latitude.

File Name: Supplementary Dataset 2

Description: Sample Metadata. This Excel file is the QIIME mapping file containing all metadata used throughout the analysis.

File Name: Supplementary Dataset 3

Description: Prevalent ‘Core’ Microbes. This Excel file summarizes prevalent microbes associated with coral microbiomes (e.g. ‘core microbiomes’, sensu lato) surveyed in this study, quantification of the effects of rarefaction depth on which OTUs are prevalent, and comparisons against two literature references. a. Graphical summary of microbial OTUs that had >70% prevalence at 1000, 5000, 10000, 15,000, or 20,000 sequences per sample. b. Machine readable data table of the prevalence and taxonomy of OTUs from panel a. c. Prevalence of microbial orders in the coral microbiome at 1000 seqs/sample, and a comparison of prevalent microbes in coral mucus with Zaneveld et al., 2016. d. Comparison with results from Apprill et al., 2016, conducted under similar rarefaction depth (10,000 seqs/sample) and prevalence threshold (50%).

File Name: Supplementary Dataset 4

Description: Beta-diversity (multivariate dissimilarities). This Excel file provides a detailed accounting of factors influencing microbial β-diversity (multivariate dissimilarities or community composition) in each compartment according to several β-diversity metrics, and across rarefaction depths. a. Factors influencing microbiome β-diversity by compartment at 1,000 sequences per sample. b. Factors influencing microbiome β-diversity by compartment at 10,000 sequences per sample. c. Factors consistently and strongly associated with coral microbiome β-diversity by compartment at 1,000 sequences per sample. To find which factors were most consistently associated with microbiome beta-diversity, we calculated factors that were a) significant b) had adjusted R2 >= 0.05 for all distance metrics analyzed. d. Summary of how taxonomic ranks structure beta-diversity. Data from a and b are combined to illustrate that more specific taxonomic ranks for corals provide more information about microbial beta-diversity than more general ranks, regardless of distance metric and rarefaction depth chosen.

File Name: Supplementary Dataset 5

Description: Alpha-diversity or richness. This Excel file provides a detailed accounting of microbiome richness. a. Results for permutational T-tests comparing coral microbiome vs. environmental community richness. Bacterial and Archaeal Diversity of Corals vs. Water and Sediment. Data reflect richness per 1000 reads of coral mucus, tissue, or sediment vs. reef water or sediment. b. Results for permutational Ttests comparing microbiome richness of corals vs. outgroups surveyed (blue corals, matt anemones, hydrozoans, etc).

File Name: Supplementary Dataset 6

Description: Microbes correlated with host and environmental parameters. This Excel file summarizes microbes that were correlated with host and environmental parameters using either Spearman or Phylogenetic GLMM analysis. a. Spearman results summary. Summary of the number of bacterial genera significantly correlated with selected host or environmental metadata in each compartment, assessed by FDRcontrolled Spearman regressions. For each factor, the Greeengenes taxonomy and R value for the top 3 genera positively or negatively correlated with that factor are listed. Additional statistical tests assess whether positive or negative associations are enriched for a given factor. b. Phylogenetic GLMM summary. Summary of numbers of genera associated with a subset of host and environmental factors, as identified in phylogenetic GLMMs. c. Full phylogenetic GLMM results. A comprehensive list of genera associated with a subset of host and environmental parameters, subdivided by tissue compartment, along with p values, estimated effect sizes, and 95% confidence intervals. For categorical data, the category value with which a microbial genus is associated is also reported.

File Name: Supplementary Dataset 7

Description: Random Forest results. This Excel file describes coral host features that can be predicted from the microbiome. To measure the strength of association between the microbiome and coral physiology, we attempted to build supervised classification models using Random Forests analysis, and then back-predict certain host features. This addresses the question: "given microbial data alone, how much can you say about the coral host?". a. Results of Random Forest models of the coral microbiome, subdivided by compartment and the host trait predicted. b. Summary of host factors that can be accurately predicted from the coral microbiome, and which compartments predict them. Raw accuracies and error ratios are presented, and results with accuracy >70% and error ratios >1.0 are highlighted.

File Name: Supplementary Dataset 8

Description: Mantel test results. This Excel file summarizes results from Mantel tests and Mantel correlograms (Methods). These permutation-based tests assess the degree of correlation between two distance matrices (e.g. geographic distance and genetic distance, etc). Here they were applied to test the extent to which host evolutionary distances corresponded to differences in microbiome composition, as reflected by betweensample beta-diversity distances. We calculated this measure for both a non-phylogenetic measure (Bray-Curtis divergences) and a phylogenetic beta-diversity distance metric (Weighted UniFrac distances).
